# Older age and frailty are the chief predictors of mortality in COVID-19 patients admitted to an acute medical unit in a secondary care setting- a cohort study

**DOI:** 10.1186/s12877-020-01803-5

**Published:** 2020-10-16

**Authors:** Rajkumar Chinnadurai, Onesi Ogedengbe, Priya Agarwal, Sally Money-Coomes, Ahmad Z. Abdurrahman, Sajeel Mohammed, Philip A. Kalra, Nicola Rothwell, Sweta Pradhan

**Affiliations:** 1 Acute Medical Unit, Fairfield General Hospital, Bury, BL9 7TD UK; 2 Department of Renal Medicine, Salford Royal NHS Foundation Trust, Salford, UK; 3 Faculty of Biology, Medicine and Health, University of Manchester, Manchester, UK

**Keywords:** COVID-19, Frailty, Mortality, Older age, Risk factors

## Abstract

**Background:**

There is a need for more observational studies across different clinical settings to better understand the epidemiology of the novel COVID-19 infection. Evidence on clinical characteristics of COVID-19 infection is scarce in secondary care settings in Western populations.

**Methods:**

We describe the clinical characteristics of all consecutive COVID-19 positive patients (*n* = 215) admitted to the acute medical unit at Fairfield General Hospital (secondary care setting) between 23 March 2020 and 30 April 2020 based on the outcome at discharge (group 1: alive or group 2: deceased). We investigated the risk factors that were associated with mortality using binary logistic regression analysis. Kaplan-Meir (KM) curves were generated by following the outcome in all patients until 12 May 2020.

**Results:**

The median age of our cohort was 74 years with a predominance of Caucasians (87.4%) and males (62%). Of the 215 patients, 86 (40%) died. A higher proportion of patients who died were frail (group 2: 63 vs group 1: 37%, *p* < 0.001), with a higher prevalence of cardiovascular disease (group 2: 58 vs group 1: 33%, *p* < 0.001) and respiratory diseases (group 2: 38 vs group 1: 25%, *p* = 0.03). In the multivariate logistic regression models, older age (odds ratio (OR) 1.03; *p* = 0.03), frailty (OR 5.1; *p* < 0.001) and lower estimated glomerular filtration rate (eGFR) on admission (OR 0.98; *p* = 0.01) were significant predictors of inpatient mortality. KM curves showed a significantly shorter survival time in the frail older patients.

**Conclusion:**

Older age and frailty are chief risk factors associated with mortality in COVID-19 patients hospitalised to an acute medical unit at secondary care level. A holistic approach by incorporating these factors is warranted in the management of patients with COVID-19 infection.

## Background

The COVID-19 pandemic is caused by the novel coronavirus (SARS- CoV-2) [1]. To date, more than 16 million cases of COVID-19 infection have been reported worldwide with the death toll currently standing above 650,000 at the time of review (July 2020). The number of positive cases and deaths are reported to be higher in the United States, Europe and Brazil compared to other regions of the world, although this has depended on the testing and reporting strategies of individual countries [2]. Understanding the epidemiology and identifying the clinical characteristics that are associated with poor outcomes can help to risk stratify patients and tailor appropriate management strategies in the approach to this pandemic. Several observational studies reported from China where the outbreak was initially reported, have helped to increase the understanding of the nature of this novel viral infection [3]. A few studies are now appearing which examine the European populations, all showing older age and a higher comorbidity burden as risk factors for mortality in COVID-19 positive patients [4–6]. Characteristics and outcome data on patients admitted to intensive care units in the United Kingdom (UK) are widely available through the Intensive Care National Audit and Research Centre (ICNARC) [7], while data from frontline acute medical units particularly at a secondary care setting is scarce. More studies are warranted in UK secondary care settings and in predominant Caucasian populations, which this study aims to address.

## Objectives

This study aims to describe and investigate the association of clinical characteristics, demographic, physical, laboratory and radiological features with outcome in patients with COVID-19 infection admitted to an acute medical unit.

## Methods

### Patient selection

This single-centre observational study was conducted on all consecutive COVID-19 positive patients admitted to the 40-bed acute medical unit (AMU) at Fairfield General Hospital, Bury, UK between 23 March 2020 and 30 April 2020. The chosen time period includes the peak incidence of reported COVID deaths in the UK (15th March to 30th April) [8]. Fairfield General Hospital is a district general hospital (secondary care centre) that is part of the Northern Care Alliance (NCA) [9]. The NCA is a group of hospitals that are situated in the North-West region of the UK, serving a population of approximately 820,000. All adult patients suspected to have symptoms and/or signs suggestive of COVID-19 and who required hospital admission had a throat swab or nose and throat swab for coronavirus identification by real-time reverse transcription polymerase chain reaction (rRT-PCR) prior to admission onto the AMU (COVID-19 cohort ward). All patients had routine blood tests and a chest X-ray at time of admission. Standard management in all patients with suspicion of bacterial chest infection included antibiotic therapy based on hospital guidelines and CURB-65 (confusion, urea, respiratory rate, blood pressure and age > 65 years) score [10] for severity of community acquired pneumonia if pneumonic changes were present on chest x-ray, plus oxygen treatment if needed. Patients with increasing oxygen requirements were assessed by a COVID team of medical specialists and appropriate management decisions were made in collaboration with an intensive care consultant regarding plans for escalation of care (mechanical ventilation, either non-invasive or invasive). Patients needing intubation and ventilation were either transferred to an intensive care unit at a tertiary care centre in the region, or level 3 care was undertaken on site.

### Data collection

**Fig. 1:**
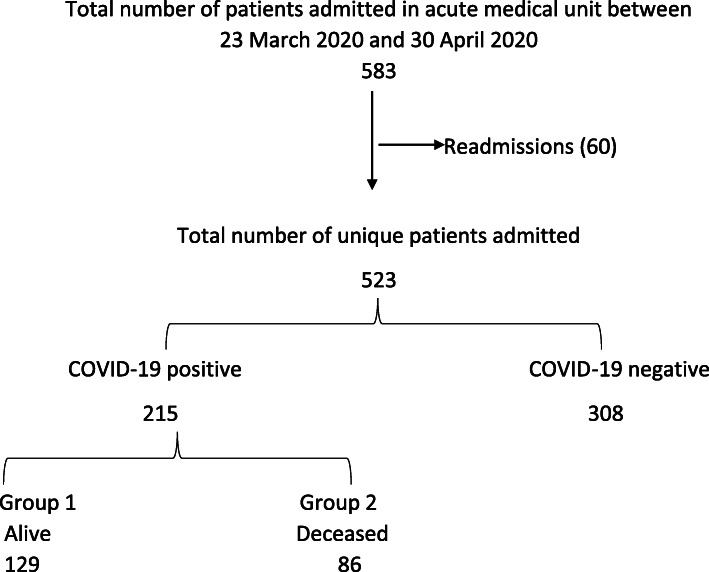
Flowchart of patient recruitment to the study

A total of 583 patients were admitted over the specified time period of which 60 were readmissions, resulting in 523 unique patient admissions. Data was collected from 215 of the 523 patients who had a positive COVID-19 rRT-PCR test result (Fig. 1). Data gathered from electronic patient records included demographics, comorbidities, smoking history, body mass index (BMI), frailty status, presenting complaint at admission, use of renin- angiotensin system inhibitor (RASi), blood parameters (full blood count, liver function tests, C-reactive protein, D-Dimer, and estimated glomerular filtration rate), radiology reports (chest X-ray) and survival outcome of hospital admission. Demographic and comorbidity data collected included age, gender, and ethnicity, history of hypertension, diabetes mellitus, cardiovascular disease, respiratory disease, chronic kidney disease, and cancer. In our study a smoking history was defined as a history of current or previous smoking irrespective of smoking pack years. RASi medications included angiotensin converting enzyme inhibitors (e.g. ramipril) and angiotensin receptor blockers (e.g. losartan). Cardiovascular disease was defined as a composite of ischemic heart disease, myocardial infarction, congestive cardiac failure and cerebrovascular accident. Respiratory disease included a composite of bronchial asthma, chronic obstructive pulmonary disease and lung fibrosis. Frailty status was determined using the clinical frailty scale (CFS) [11, 12]. Any patient with a score of five and above on the CFS was defined as being frail, which also included seven patients below the age of 65 years based on clinician assessment.

### Statistical analysis

Patients were split into two groups based on the survival outcome of hospital admission (group: 1 alive on discharge and group 2: deceased) and analysed. Any patient who was discharged on end-of-life care to a hospice or nursing home was included in group 2 as all these patients died within a week of discharge (12 patients).

In the descriptive analysis of the data, continuous variables (age, body mass index and blood parameters) were expressed as median (interquartile range) after checking the normality of the distribution and the *p*-values were derived using Mann-Whitney U test. The categorical variables (gender, race (Caucasian or other), and comorbidities) were expressed as number (%), and *p*-values were derived using the Chi-square test. A *p*-value < 0.05 (2-tailed) was considered statistically significant throughout the analysis.

Univariate and multivariate binary logistic regression models were used to study risk factors that are predictors for mortality. The results from the models were expressed as odds ratio (95% confidence interval) and a *p*-Value for statistical significance. Three multivariate (MV) models were developed by incorporating variables that were statistically significant in the univariate model. MV model-1 included clinical characteristics with the complete dataset, MV model-2 included laboratory characteristics and the MV model-3 included all the variables that were significant in the univariate model. Survival outcome for all patients was also followed up from the date of admission until an arbitrary study end-point date, 12 May 2020, which was used to generate the Kaplan- Meier (KM) curves and Cox-regression models. The proportional hazard assumption for the Cox- model was examined and met by plotting the log-minus-log survival curves and survival times against cumulative survival. All analyses were carried out using SPSS Version 23 licenced to the University of Manchester. The study was registered the Northern Care Alliance Research and Innovation department (ID: P20HIP20). As this was an observational study with complete anonymization of patient details, the need for individual consent was waived.

## Results

In our cohort of COVID-19 positive patients 40% (86/215) died. Our cohort had a predominance of Caucasians (87.4%) and had a median age of 74 years. Patients who died (group 2) were older (80 vs 67 years, *p* < 0.001), had a higher proportion of care home residents (43 vs 18%, *p* < 0.001), and were more frail (62.7 vs 37.3%, *p* < 0.001).

**Fig. 2:**
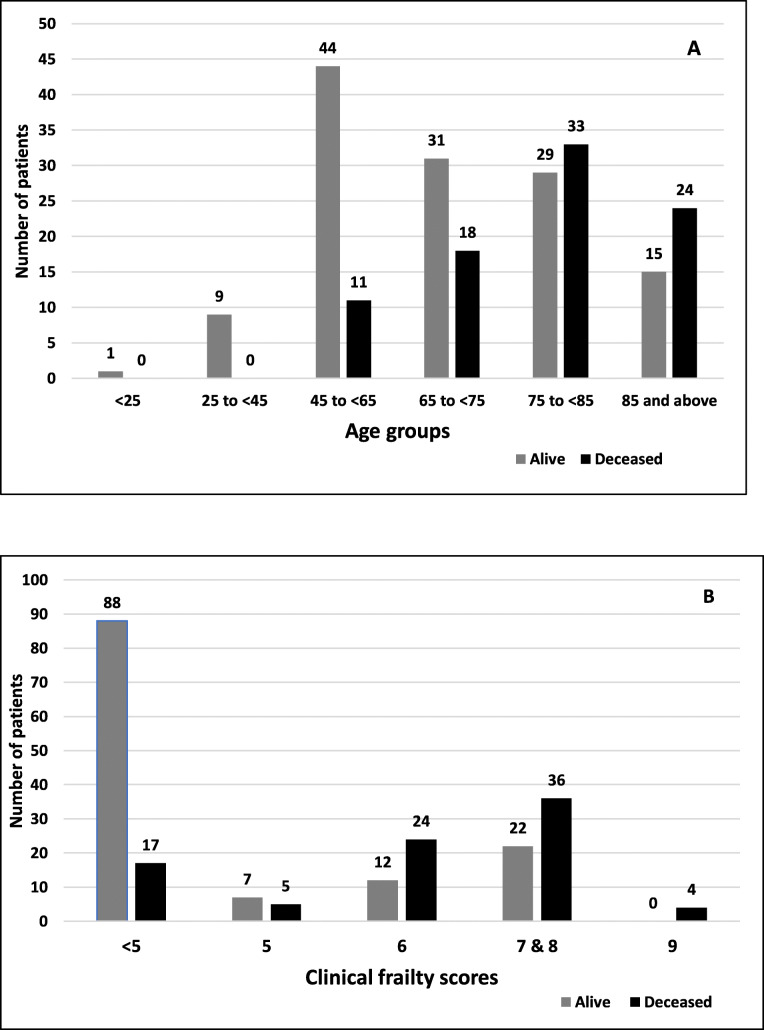
Distribution of outcomes based on age groups (**a**) and clinical frailty scores (**b**)

Figure 2 shows the influence of age and frailty upon mortality. Only 17% of patients aged < 65 years died, whereas mortality in the 65–75 years, 75–85 years and > 85 years groups was 37, 53 and 62% respectively. The frailty scores indicated that only 16% of those with a score of < 5 died, whereas mortality in those with frailty scores of 5, 6, 7/8 combined and 9 were 42, 67, 62 and 100%, respectively.

Surviving patients (group 1) had a higher body mass index (BMI) (29.4 vs 26 kg/m^2^, *p* < 0.001) but a smaller proportion had cardiovascular disease compared to group 2 (33.3 vs 58.1, *p* < 0.001). Respiratory diseases were also more prevalent amongst group 2 patients (38 vs 25%, *p* = 0.03). In all patients shortness of breath was the chief presenting complaint on hospital admission (80%) followed by cough (57%) and fever (46%). Other presenting features noted in a minority of patients included gastro-intestinal symptoms (diarrhoea, vomiting and abdominal pain), chest pain, confusion, lethargy and feeling generally unwell. No difference was observed in patients taking RASi or immunosuppressant medications between the two groups. Median duration of hospital stay was 5 days which was similar in both groups. Of the total patients, 24 (11.2%) patients who received mechanical ventilation the mortality rate was 50%; 7.5% received non-invasive ventilation and 3.7% underwent intubation and ventilation (Table 1).

**Table 1:** Clinical characteristics of COVID-19 positive patients at hospital admission

Characteristics	Total 215	Group-1 Alive 129	Group-2 Deceased 86	*P*-Value (Alive vs Deceased)
Age	74 (60–82)	67 (57–79)	80 (73–86)	**< 0.001**
Gender, Male	133 (61.9)	82 (63.5)	51 (59.3)	0.53
Ethnicity, Caucasian	188 (87.4)	111 (86)	77 (89.5)	0.45
Care home resident	60 (27.9)	23 (17.8)	37 (43)	**< 0.001**
Frailty	110 (51.2)	41 (37.3)	69 (62.7)	**< 0.001**
Smoking	120 (55.8)	65 (50.4)	55 (63.9)	**0.05**
Weight	78 (67–92)	84.5 (71.6–100)	70 (63–84)	**< 0.001**
BMI, kg/m^2^	28 (24–32)	29.4 (26–34)	26 (23–29)	**< 0.001**
Hypertension	114 (53)	62 (48.1)	52 (60.5)	0.07
Diabetes mellitus	65 (30.2)	42 (32.5)	23 (26.7)	0.36
CVD	93 (43.3)	43 (33.3)	50 (58.1)	**< 0.001**
IHD and MI	53 (24.7)	28 (21.7)	25 (29.1)	0.22
CCF	39 (18.1)	15 (11.6)	24 (27.9)	**0.002**
CVA	30 (14)	11 (8.5)	19 (22.1)	**0.005**
CKD (stage 3–5)	42 (19.5)	24 (18.6)	18 (20.9)	0.67
Cancer	19 (8.8)	13 (10.1)	6 (7)	0.43
Respiratory diseases	65 (30.2)	32 (24.8)	33 (38.4)	**0.03**
On RASi treatment	54 (25)	37 (28.7)	17 (19.8)	0.09
Immunosuppression	12 (5.6)	6 (4.6)	6 (7)	0.47
Trial participation	39 (18.1)	27 (20.9)	12 (13.9)	0.19
Presenting complaint				
Shortness of breath	172 (80)	100 (77.5)	72 (83.7)	0.27
Fever	98 (45.6)	66 (51.2)	32 (37.2)	0.08
Cough	122 (56.7)	92 (71.3)	30 (34.8)	**< 0.001**
Mechanical ventilation	24 (11.2)	12 (9.3)	12 (13.9)	0.20
Non-invasive ventilation	16 (7.5)	9 (7.0)	7 (5.4)	0.75
Intubation & Ventilation	8 (3.7)	3 (3.5)	5 (5.8)	0.19
Hospital inpatient (days)	5 (2–10)	5 (2–10)	5 (3–9)	0.47

Continuous variables are expressed as median (interquartile range) and *p*-Value by Man-Whitney U test

Categorical variables are expressed as number (%) and *p*-Value by Chi-square test

Weight missing in 12/215, BMI missing in 15/215

*BMI* Body mass index, *CVD* Cardio vascular disease; includes at least one of the following- ischemic heart disease (*IHD*), myocardial infarction (*MI*), congestive cardiac failure (*CCF*), cerebrovascular accident (*CVA*), *CKD* Chronic kidney disease, *RASi* Renin-angiotensin system inhibitors. Respiratory diseases include a composite of asthma, chronic obstructive pulmonary disease and pulmonary fibrosis

On evaluation of the laboratory characteristics (Table 2), patients in group 2 had a lower lymphocyte count and consequently a higher neutrophil to lymphocyte ratio (9 vs 6, *p* = 0.004). No significant difference in the liver function tests was seen between the groups. Group 2 patients had a lower albumin (median 29 vs 31 g/L, *p* = 0.01), a higher C-reactive protein (CRP) (median 123 vs 90 mg/L, *p* = 0.009) and d-dimer (775 vs 559 ng/mL, *p* = 0.04), although a d-dimer test result was not routinely performed at our centre and was performed in only 15 patients. Group 2 patients had a significantly lower eGFR on admission (49 vs 77 mL/min/1.73m^2^, *p* < 0.001) and a higher proportion had acute kidney injury at presentation (47 vs 19%, *p* < 0.001). Chest-X ray features did not significantly differ between the groups with 80% overall having changes suggestive of COVID-19 and 56% having bilateral infiltrates.

**Table 2:** Laboratory and radiological characteristics of COVID-19 positive patients at hospital admission

Characteristics	Total 215	Group-1 Alive 129	Group-2 Deceased 86	*p*-Value (Alive vs Deceased)
Haemoglobin, g/L	133 (120–146)	134 (122–148)	129 (118–143)	0.08
Neutrophil count, × 10^9^/L	6 (4–9)	6 (4–8)	7 (4–9)	**0.02**
Lymphocyte count, × 10^9^/L	0.9 (0.6–1.3)	0.9 (0.6–1.4)	0.8 (0.5–1.2)	**0.03**
New lymphopenia	85 (39.5)	49 (37.9)	36 (41.9)	0.57
Neutrophil: lymphocyte ratio	7 (4–13)	6 (4–11)	9 (5–18)	**0.004**
Platelet count, ×10^9^/L	217 (161–270)	223 (162–270)	210 (155–265)	0.23
Albumin, g/L	30 (27–34)	31 (28–35)	29 (26–32)	**0.01**
Bilirubin, umol/L	12 (8–17)	12 (8–18)	11 (8–16)	0.86
Alanine transaminase, U/L	27 (18–45)	28 (18–48)	27 (19–39)	0.66
Alkaline phosphatase, U/L	81 (63–109)	79 (62–107)	85.5 (65–109)	0.35
C-reactive protein, mg/L	107 (56–177)	90 (41–164)	123 (72–189)	**0.009**
D-Dimer, ng/mL	610 (297–809)	559 (412–748)	775 (701–848)	**0.04**
eGFR, mL/min/1.73m^2^	67 (42–90)	77 (56–90)	48.5 (28–74)	**< 0.001**
Acute kidney injury (any stage)	65 (30.2)	25 (19.4)	40 (46.5)	**< 0.001**
Chest X-Ray report				
Suggestive of COVID-19	166 (79.8)	98 (77.8)	68 (82.9)	0.37
Bilateral infiltrates	117 (56.2)	67 (53.2)	50 (61)	0.27
Unilateral consolidation	48 (23.1)	31 (24)	17 (19.8)	0.46

Continuous variables are expressed as median (interquartile range) and *p*-Value by Man-Whitney U test. Categorical variables are expressed as number (%) and *p*-Value by Chi-square test

Missing albumin, alanine transaminase, alkaline phosphatase, bilirubin in 32/215 patients. Missing c-reactive protein in 5/215 patients. D-Dimer available only from 15 patients. Missing chest X-ray report- 7 patients

*eGFR* Estimated glomerular filtration rate calculated by CKD-EPI equation, *U/L* Units/litre

Table 3 illustrates the binary logistic regression models. In the univariate binary logistic regression model, several characteristics including older age, care home residence, frailty, positive smoking history, lower weight and BMI, comorbidities (cardiovascular & respiratory), acute kidney injury on admission, a higher neutrophil count, lower lymphocyte count, higher CRP and lower eGFR were noted to be significant predictors of mortality. In MV model-1 older age (OR:1.03; 95%CI: 1.01–1.06; *p* = 0. 03) and frailty (OR:5.1; 95%CI: 2.3–11.6; *p* < 0.001) were noted to be significant predictors of mortality. Furthermore, in MV model-2 which included all the significant biochemical variables, a lower eGFR on admission (OR:0.98; 95%CI: 0.96–0.99; *p* = 0.01) was observed as a significant predictor of mortality. Frailty emerged as the only significant predictor for mortality in the MV model-3 (OR:4.3; 95%CI: 1.7–10.8; *p* = 0.002) (Table 4).

**Table 3:** Predictors of mortality in COVID-19 positive patients by binary logistic regression models (univariate and multivariate model 1&2)

Characteristics	Univariate model OR (95% CI)	*P*-Value	Multivariate model 1 OR (95% CI)	*P*-Value	Multivariate model 2 OR (95% CI)	*P*-Value
Age	1.06 (1.03–1.09)	**< 0.001**	1.03 (1.01–1.06)	**0.03**		
Gender, Male	0.83 (0.48–1.46)	0.53				
Ethnicity, Caucasian	1.38 (0.59–3.20)	0.45				
Care home resident	3.48 (1.57–6.47)	**< 0.001**	1.12 (0.52–2.40)	0.77		
Frailty	8.71 (4.56–16.6)	**< 0.001**	5.1 (2.3–11.60)	**< 0.001**		
Smoking	1.75 (0.99–3.05)	**0.05**	1.57 (0.81–3.01)	0.18		
Weight	0.96 (0.95–0.98)	**< 0.001**				
BMI	0.90 (0.86–0.95)	**< 0.001**				
Hypertension	1.60 (0.95–2.87)	0.07				
Diabetes mellitus	0.76 (0.41–1.38)	0.36				
CVD	2.77 (1.58–4.88)	**< 0.001**	1.20 (0.61–2.40)	0.59		
IHD/MI	1.47 (0.79–2.76)	0.22				
CCF	2.94 (1.43–6.02)	**0.003**				
CVA	3.04 (1.36–6.77)	**0.006**				
CKD (stage 3–5)	1.15 (0.58–2.29)	0.67				
Cancer	0.67 (0.24–1.83)	0.44				
Respiratory diseases	1.88 (1.05–3.40)	**0.035**	1.51 (0.75–3.06)	0.24		
On RASi treatment	0.61 (0.32–1.17)	0.14				
Immunosuppression	1.50 (0.48–4.90)	0.47				
Haemoglobin	0.99 (0.97–1.00)	0.16				
Neutrophil count	1.08 (1.01–1.14)	**0.02**			0.95 (0.85–1.04)	0.28
Lymphocyte count	0.59 (0.36–0.98)	**0.04**			1.19 (0.66–2.10)	0.55
Neutrophil: lymphocyte ratio	1.05 (1.01–1.08)	**0.002**			1.05 (0.99–1.11)	0.06
Platelet count	0.99 (0.96–1.00)	0.31				
Albumin	1.00 (0.98–1.02)	0.99				
Bilirubin	1.01 (0.98–1.04)	0.44				
Alanine transaminase	1.00 (0.99–1.00)	0.45				
Alkaline phosphatase	1.01 (0.99–1.01)	0.27				
C-reactive protein	1.01 (1.0–1.010)	**0.010**			1.0 (0.99–1.00)	0.26
eGFR	0.97 (0.96–0.98)	**< 0.001**			0.98 (0.96–0.99)	**0.01**
Acute kidney injury	3.60 (1.96–6.65)	**< 0.001**			1.78 (0.80–3.99)	0.16

Multivariate model 1: adjusted for age, care home resident, frailty, smoking, CVD, and respiratory diseases

Multivariate model 2: adjusted for neutrophil count, lymphocyte count, neutrophil: lymphocyte ratio, C-reactive protein, eGFR, and acute kidney injury

*BMI* Body mass index, *CVD* Cardio vascular disease; includes at least one of the following- ischemic heart disease (*IHD*), myocardial infarction (*MI*), congestive cardiac failure (*CCF*), cerebrovascular accident (*CVA*), *CKD* Chronic kidney disease, *RASi* Renin-angiotensin system inhibitors. Respiratory diseases include a composite of asthma, chronic obstructive pulmonary disease and pulmonary fibrosis. *eGFR* Estimated glomerular filtration rate calculated by CKD-EPI equation, *OR* Odds ratio, *CI* Confidence interval

**Table 4:** Predictors of mortality in COVID-19 positive patients by binary logistic regression model (multivariate model 3)

Characteristics	Multivariate model 3 OR (95% CI)	*P*-Value
Age	1.03 (0.99–.07)	0.10
Care home resident	0.69 (0.28–1.68)	0.42
Frailty	4.3 (1.71–10.76)	**0.002**
Smoking	1.64 (0.75–3.58)	0.21
BMI	0.96 (0.91–1.03)	0.29
CVD	1.68 (0.77–3.68)	0.19
Respiratory diseases	1.25 (0.57–2.78)	0.57
Neutrophil: lymphocyte ratio	1.02 (0.98–1.06)	0.25
C-reactive protein	1.01 (1–1.01)	0.07
eGFR	0.99 (0.97–1.01)	0.32
Acute kidney injury	1.6 (0.63–4.09)	0.31

Multivariate model 3: adjusted for age, care home resident, frailty, smoking, BMI, CVD, respiratory diseases, neutrophil: lymphocyte ratio, C-reactive protein (CRP), eGFR, and acute kidney injury. Model did not include 18 patients without BMI and CRP values

*BMI* Body mass index, *CVD* Cardio vascular disease; includes at least one of the following- ischemic heart disease (*IHD*), myocardial infarction (*MI*), congestive cardiac failure (*CCF*), cerebrovascular accident (*CVA*), Respiratory diseases include a composite of asthma, chronic obstructive pulmonary disease and pulmonary fibrosis. *eGFR* Estimated glomerular filtration rate calculated by CKD-EPI equation, *OR* Odds ratio, *CI* Confidence interval

**Fig. 3:**
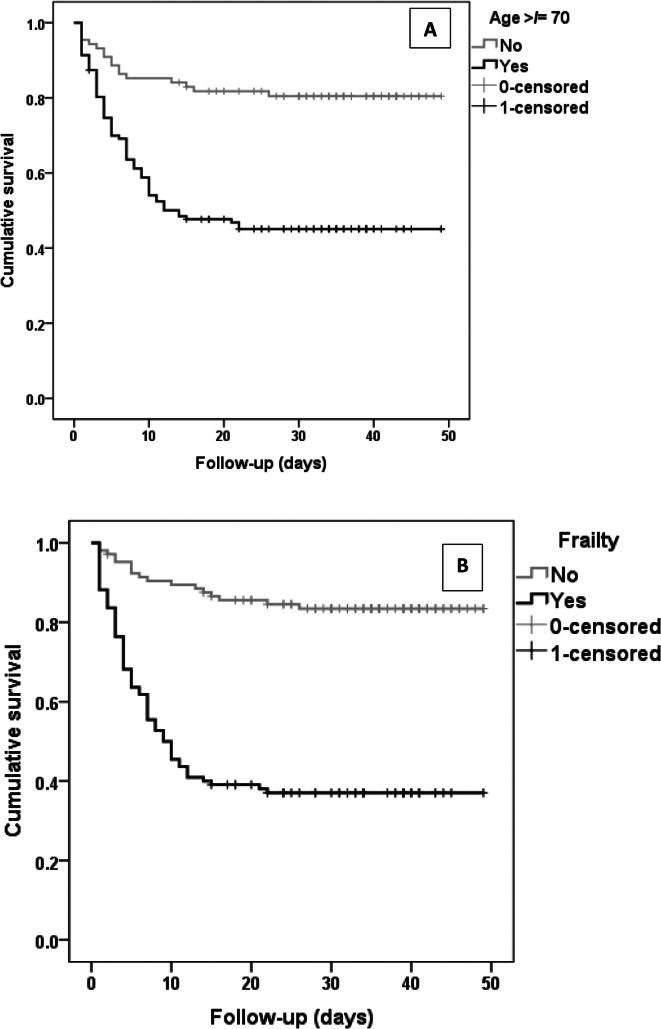
Kaplan-Meier curves for mortality based on age category (**a**) and frailty status (**b**)

During the follow-up time period until 12/05/2020, two additional deaths were recorded. The KM curves developed from the follow-up data showed a significant difference in outcomes in older aged and frail patients (log-rank *p* < 0.001) (Fig. 3). The Cox- regression analysis provided similar observations as the logistic regression models, with older age (Hazard ratio (HR):1.03; 95%CI:1.01–1.05; *p* = 0.01) and frailty (HR:3.45; 95%CI: 1.76–6.79; *p* < 0.001) as significant risk factors associated with mortality (supplementary tables 1 & 2).

## Discussion

This is an observational study of COVID-19 positive patients admitted to an acute medical unit in a district general hospital (secondary care setting). The study describes the clinical characteristics of COVID-19 positive patients at presentation and investigates the risk factors associated with mortality following hospital admission.

The mortality rate (proportion of the total) of our cohort of hospitalised COVID-19 positive patients was 40%. The age standardised mortality rate for COVID-19 in the Manchester area was reported as 55% in a similar time period by the Office of the National Statistics (ONS) [13]. A higher mortality figure reported in the ONS data is likely to be due to inclusion of deaths from all the care homes in the region and the intensive care units, which our study did not encompass. We observed an increasing trend in mortality with advancing age which was in line with the national statistics, possibly due to increase in the comorbid burden and altered immune response with advancing age [14, 15]. We did not observe any significant difference in outcome associated with variances in gender and ethnicity, but our studied population was predominantly Caucasian (87.4%). It has been reported that men are more at risk of death than women in a small cohort of COVID-19 positive patients in China involving 43 patients [16]. We found that deaths were proportionately higher in care home residents, who are generally more frail than patients residing in their own homes. More than 50% of our cohort were frail and there was a higher percentage of frailty in the deceased group (63 vs 37%, *p* < 0.001). The National Institute for Health and Care Excellence (NICE) published a guideline on March 2020 to use the Clinical Frailty Scale as available from the NHS Specialised Clinical Frailty Network, for all adult hospital admissions to assess frailty irrespective of COVID-19 status as a part of holistic assessment [17]. The NHS Specialised Clinical Frailty Network recommends that Clinical Frailty Scale can be undertaken by any trained healthcare professional (doctor, nurse, health care assistant, therapist etc.) by asking the patient or their carer/next of kin/paramedics/care home staff what their/the patient’s capability was 2 weeks prior to current admission [18]. In our real world retrospective observational study, we have collected CFS from electronic patient records as recorded by trained clinical staff (doctors and nurses) on hospital admission.

In our cohort 53% had a history of hypertension, 30% had diabetes, and 30 and 43% had at least one respiratory and cardiovascular disease, respectively. All these comorbidities were noted to be risk factors associated with poor outcomes in patients with COVID-19 infection in a meta-analysis of six studies with a total of 1558 patients [19]. Although the presenting symptoms of shortness of breath and fever were similar between the groups, cough was less reported (35 vs 71%, *p* < 0.001) in deceased patients, which supports the speculation that lack of a cough reflex can promote worse infection in elderly frail patients [19].

In the univariate logistic regression models several clinical characteristics were observed to show significant association with mortality. Older age showed a significant association with mortality in our cohort (OR 1.06; *p* < 0.001). Old age as a risk factor for mortality has been reported in a Chinese cohort with a median age of 67 years [20]. An association of smoking with poor outcome (OR 1.75; *P* = 0.05) has been variably reported in other observational studies [21, 22]. The risk of death within 15 days of hospital admission for COVID-19 infection was found to be higher in elderly patients with a history of smoking and underlying respiratory comorbidities [23].

In our study, diabetes mellitus and hypertension were not significant predictors of mortality. Both hypertension and diabetes have been shown to be associated with increased mortality in two separate meta-analyses [24, 25], but the strength of the effect was weak with older age (> 55 years). The mean age of most of the studies included in these meta-analyses was less than 60 years compared to the median age of our cohort (74 years). Also, our study showed that a lower BMI was a risk factor for mortality (OR 0.90; *p* < 0.001), although the median BMI of survivors was in the normal (not obese) range. The association of obesity with severity of COVID-19 illness has been demonstrated in an observational study in China of 383 hospitalised patients, but the mean age of this cohort was less than 50 years [26]. The influence of older age and frailty on poorer nutrition and reduced BMI could have influenced these observations in our cohort.

A history of cardiovascular disease (OR 2.77; *p* < 0.001) and respiratory disease (OR 1.88; *p* < 0.035) showed positive association with mortality in accordance with studies reported in other regions [27, 28]. Several pathophysiological mechanisms have been proposed that can link increased mortality in COVID-19 infected patients with cardiovascular and respiratory co-morbidities including predisposition to acute respiratory distress syndrome and myocardial injury, although the evidence is still evolving [29]. Although there has been much debate regarding the impact of RASi treatment on poor outcome in COVID-19 infected patients, in our cohort, in which 25% were receiving RASi treatment, a significant association was not observed (OR 0.61; *P* = 0.14) [30, 31]. Among the laboratory variables a lower lymphocyte count (OR 0.59; *p* = 0.04) and a higher neutrophil: lymphocyte ratio (OR 1.05; *p* = 0.002) were predictors of mortality which is similar to findings in other observational studies [32]. Dysregulation of the immune response resulting in reduced CD4+ helper T lymphocytes has been observed in patients with COVID-19 infection, more so in severe cases [33].

A lower eGFR on admission, and also acute kidney injury, proved to be risk factors associated with mortality, and low eGFR was independently associated in a multivariate model (OR 0.98; *p* = 0.01), an observation reported in a recent study on the influence of kidney disease on mortality in patients with COVID-19 [34].

In addition to eGFR, the multivariate models showed older age and frailty as a significant risk factors associated with mortality in COVID-19 positive patients. The influence of frailty (frailty score of 5 or more) upon mortality outweighed that of age in our cohort (MV model 3; OR 4.3; *p* = 0.002 vs OR 1.03; *p* = 0.10), possibly due to the distribution of frailty which affected patients as young as 65 years of age. Several studies have reported age as a risk factor associated with mortality [35–37], and our findings are also supported by the recently published multicentre study in the United Kingdom showing a positive association between frailty and mortality [38].

In our centre, the escalation of care to mechanical ventilation for deteriorating COVID-19 patients was largely determined by the patient’s functional status using clinical frailty score, and comorbid burden by a COVID team (doctors at consultant and senior registrar level in chest or general or intensive care medicine) in liaison with an intensive care specialist at the tertiary care referral centre. However, this approach was individualised on a case-by-case basis taking into account the severity of the clinical presentation. Both the patient and family members were fully involved in the decision-making process wherever possible.

This study could not include patients who were directly transferred to the intensive care unit for mechanical ventilation from the emergency department, thereby missing the opportunity to capture the characteristics and outcomes of patients who were critically sick at initial presentation. However, the epidemiology of this group of patients is well presented in the ICNARC data. The study is also limited by the single centre observational nature of the study methodology.

## Conclusion

In conclusion, our study highlights that in addition to comorbid burden, older age and frailty were the chief risk factors that were associated with mortality in patients hospitalised in a secondary care acute medical unit. Health care providers need to be increasingly aware of the impact of age and frailty on survival and should institute a holistic approach in the management of COVID-19 positive patients in liaison with the patient, family members and specialists to achieve the most appropriate care for patients with this novel infection.

## Supplementary information

**Additional file 1: Supplementary table 1.** Association between risk factors and mortality in COVID-19 positive patients by cox-regression models (univariate and multivariate model 1&2). **Supplementary table 2.** Association between risk factors and mortality in COVID-19 positive patients by cox-regression model (multivariate model 3).

## Data Availability

The datasets used and/or analysed during the current study are available from the corresponding author on reasonable request.

## Abbreviations

AMU: Acute medical unit
BMI: Body mass index
CFS: Clinical frailty scale
CRP: C-reactive protein
eGFR: Estimated glomerular filtration rate
HR: Hazard ratio
ICNARC: Intensive Care National Audit and Research Centre
KM: Kaplan-Meier
MV: Multivariate
NCA: Northern Care Alliance
OR: Odds ratio
RASi: Renin-angiotensin system inhibitors
rRT-PCR: Real-time reverse transcription polymerase chain reaction
