# Supplementary material for: Older age and frailty are the chief predictors of mortality in COVID-19 patients admitted to an acute medical unit in a secondary care setting- a cohort study
Source: BMC Geriatr. 2020 Oct 16;20:409. doi: 10.1186/s12877-020-01803-5 (PMC7563906; doi:10.1186/s12877-020-01803-5)
Supplement: Supplementary file 1 — Additional file 1: Supplementary table 1. Association between risk factors and mortality in COVID-19 positive patients by cox-regression models (univariate and multivariate model 1&2). Supplementary table 2. Association between risk factors and mortality in COVID-19 positive patients by cox-regression model (multivariate model 3). [file 12877_2020_1803_MOESM1_ESM.docx]

Supplementary table 1 Association between risk factors and mortality in COVID-19 positive patients by cox-regression models (univariate and multivariate model 1&2)

| Characteristics | Univariate model  HR (95% CI) | p-Value | Multivariate model 1  HR (95% CI) | p-Value | Multivariate model 2  HR (95% CI) | p-Value |
| --- | --- | --- | --- | --- | --- | --- |
| Age | 1.04 (10.2-10.6) | **<0.001** | 1.03 (1.01-1.05) | **0.01** |  |  |
| Gender, Male | 0.92 (0.60-1.42) | 0.72 |  |  |  |  |
| Ethnicity, Caucasian | 1.16 (0.58-2.32) | 0.66 |  |  |  |  |
| Care home resident | 2.43 (1.58-3.73) | **<0.001** | 0.74 (0.43-1.25) | 0.26 |  |  |
| Frailty | 5.52 (3.23-9.42) | **<0.001** | 3.45 (1.76-6.79) | **<0.001** |  |  |
| Smoking | 1.60 (1.03-2.48) | **0.04** | 1.88 (1.12-3.16) | **0.02** |  |  |
| Weight | 0.97 (0.96-0.98) | **<0.001** |  |  |  |  |
| BMI | 0.92 (0.89-0.96) | **<0.001** | 0.99 (0.94-1.03) | 0.65 |  |  |
| Hypertension | 1.52 (0.98-2.32) | 0.06 |  |  |  |  |
| Diabetes mellitus | 0.82 (0.51-1.32) | 0.43 |  |  |  |  |
| CVD | 2.15 (1.40-3.31) | **<0.001** | 1.12 (0.69-1.83) | 0.65 |  |  |
| IHD/MI | 1.40 (0.88-2.23) | 0.16 |  |  |  |  |
| CCF | 2.02 (1.26-3.23) | **0.003** |  |  |  |  |
| CVA | 2.26 (1.35-3.76) | **0.002** |  |  |  |  |
| CKD (stage 3-5) | 1.23 (0.73-2.07) | 0.43 |  |  |  |  |
| Cancer | 0.75 (0.33-1.72) | 0.50 |  |  |  |  |
| Respiratory diseases | 1.56 (1.01-2.42) | **0.043** | 1.12 (0.69-1.82) | 0.64 |  |  |
| On RASi treatment | 0.73 (0.43-1.24) | 0.24 |  |  |  |  |
| Immunosuppression | 1.45 (0.63-3.33) | 0.38 |  |  |  |  |
| Haemoglobin | 0.99 (0.98-1.01) | 0.08 |  |  |  |  |
| Neutrophil count | 1.05 (1.01-1.08) | **0.015** |  |  | 0.98 (0.93-1.03) | 0.47 |
| Lymphocyte count | 0.66 (0.44-1.00) | 0.05 |  |  |  |  |
| Neutrophil: lymphocyte ratio | 1.02 (1.01-1.04) | **<0.001** |  |  | 1.01 (0.99-1.03) | 0.12 |
| Platelet count | 0.99 (0.99-1.00) | 0.27 |  |  |  |  |
| Albumin | 1.00 (0.98-1.02) | 0.89 |  |  |  |  |
| Bilirubin | 1.01 (0.99-1.03) | 0.24 |  |  |  |  |
| ALT | 1.00 (0.99-1.00) | 0.17 |  |  |  |  |
| ALP | 1.00 (0.99-1.01) | 0.33 |  |  |  |  |
| C-reactive protein | 1.01 (1.01-1.02) | **0.001** |  |  | 1.01 (1.00-1.01) | **0.03** |
| eGFR | 0.98 (0.97-0.98) | **<0.001** |  |  | 0.98 (0.97-0.99) | **0.003** |
| Acute kidney injury | 2.61 (1.71-3.99) | **<0.001** |  |  | 1.49 (0.87-2.5) | 0.14 |

BMI-body mass index, CVD- cardio vascular disease; includes at least one of the following- ischemic heart disease (IHD), myocardial infarction (MI), congestive cardiac failure (CCF), cerebrovascular accident (CVA), CKD- chronic kidney disease, RASi-renin-angiotensin system inhibitors . Respiratory diseases include a composite of asthma, chronic obstructive pulmonary disease and pulmonary fibrosis. eGFR - estimated glomerular filtration rate calculated by CKD-EPI equation. HR-Hazard ratio, CI-confidence interval

Multivariate model 1: Included age, care home resident, frailty, smoking, BMI, CVD, and respiratory diseases.

Multivariate model 2: Included neutrophil count, neutrophil: lymphocyte ratio, C-reactive protein, eGFR, and acute kidney injury.

Supplementary table 2 Association between risk factors and mortality in COVID-19 positive patients by cox-regression model (multivariate model 3)

| Characteristics | Multivariate model 3  HR (95% CI) | p-Value |
| --- | --- | --- |
| Age | 1.03 (0.99-1.06) | 0.06 |
| Care home resident | 0.61 (0.35-1.07) | 0.08 |
| Frailty | 3.46 (1.76-6.81) | **<0.001** |
| Smoking | 1.61 (0.93-2.77) | 0.09 |
| BMI | 0.98 (0.94-1.03) | 0.43 |
| CVD | 1.34 (0.80-2.24) | 0.27 |
| Respiratory diseases | 1.15 (0.70-1.89) | 0.58 |
| Neutrophil: lymphocyte ratio | 1.00 (0.98-1.01) | 0.95 |
| C-reactive protein | 1.01 (1.00-1.01) | **0.001** |
| eGFR | 0.99 (0.98-10.01) | 0.13 |
| Acute kidney injury | 1.20 (0.67-2.14) | 0.53 |

BMI-body mass index, CVD- cardio vascular disease; includes at least one of the following- ischemic heart disease (IHD), myocardial infarction (MI), congestive cardiac failure (CCF), cerebrovascular accident (CVA), Respiratory diseases include a composite of asthma, chronic obstructive pulmonary disease and pulmonary fibrosis. eGFR - estimated glomerular filtration rate calculated by CKD-EPI equation. HR-Hazard ratio, CI-confidence interval

Multivariate model 3: adjusted for age, care home resident, frailty, smoking, BMI, CVD, respiratory diseases, neutrophil: lymphocyte ratio, C-reactive protein, eGFR, and acute kidney injury. Model did not include 18 patients without BMI and CRP values.
